# Medical Expert and Medical Evidence

**Published:** 1875-02

**Authors:** M. A. McClelland

**Affiliations:** Knoxville, Ill.


### Chicago Medical Journal.

A MONTHLY RECORD OF

*Medicine, Surgery and the Collateral Sciences.*

EDITED BY J. ADAMS ALLEN, M.D., LL.D.: AND WALTER HAY, M.D.

VOL. XXXII. — FEBRUARY, 1875. — NO. 2.

### Original Communications.

ARTICLE I.

### THE MEDICAL EXPERT AND MEDICAL EVIDENCE.

BY M. A. McCLELLAND, M.D., KNOXVILLE, ILL.

(Continued from page 28.)

WHO ARE MEDICAL EXPERTS?

This is a vexatious question. As questions in medicine are the most common, and frequently the most important, involving matters of property, reputation, and, indeed, life and death, its proper solution is of the greatest importance. In the first place, Who are physicians? In some countries the question is answered by the statutes. In the United States, "proof that one practices as a physician is *prima facie* evidence of his professional character," (*Sutton* v. *Tracy*, 1 Mich. 243), but only as "to the business in which the plaintiff was engaged, rather than to the degree of skill with which he exercised it." That a person practices medicine is no proof of *skill* is now being recognized by statutory enactments in various States, requiring persons practicing medicine to be in possession of some evidence of their qualification, in the form of a diploma or license from an examining board of physicians. It will be seen, however, from what has been before said, that the possession of these evidences of capability to practice does not confer *ability* to act creditably as an expert.

"In forensic-psychology an expert must be skilled (1) in law, sufficient to determine what is the 'responsibility' which is to be the object of the contested capacity; (2) psychology, so as to be able to speak analytically as to the properties of the human mind; (3) medicine, so far as concerns the treatment of the insane, so as to speak inductively on the same subject. If either of these factors is wanting, a witness cannot be technically an expert." (Wharton and Stille, Med. Jurisp., Book 1, § 275.) This is a very high standard, and practically is not observed. Courts have modified it, and in doing so have fallen into the other extreme, permitting many persons to testify who are not competent to do so. They ignore the fact that he only is an *expert* who is *instructed* in any science, art or trade, by *study* and *experience.* See *Fairchild* v. *Blascomb*, 35 Vt. 390, where it was held that *nurses*, accustomed to attend the sick, were *competent* experts in respect to mental conditions. In *State* v. *Hinkle*, 6 Iowa, 380, it was held that an ordinary physician, at least not an analytical chemist, was capable of analyzing the contents of a stomach of one supposed to be poisoned. Similar rulings may be found in other States. Why should they be "highly regarded"?

In cases of insanity, however, the rule for requiring that experts shall have made the subject of mental disease a special study, is pretty generally insisted upon, especially in the higher courts. In many of our States, patients, supposed to be insane, cannot be committed to hospitals or other places of confinement, without a preliminary examination before one of the lower courts. In this examination, one or more physicians are summoned to appear to act as juror or witness. Ordinarily physicians are exempt from jury duty; in this case the duty is imperative. The necessity for this judicial investigation, *ab initio*, has arisen from a suspicion that sane persons have been thus imprisoned, so that some dishonest relative might be better able to carry on some scheme of iniquity. This suspicion is kept alive in the minds of the community by such sensational efforts as are manifested in the "*Terrible Temptation*" of Charles Reade. I was about to mention, also, "*Behind the Bars*," but I am not acquainted with its tendencies. Physicians will readily call to mind the "sensation" created a few years ago by the publication of a book that purported to give the history of a number of persons unjustly confined in one of our State institutions. It is needless to state that the charges made in it had no foundation in fact.

The true preparation for this duty, as Dr. Ray remarks, (Med. Jurisp. of Insanity), "lies in observation and study of mental phenomena, as strikingly exhibited in real life." A large acquaintance with mental phenomena, in a sound as well as unsound state, a comprehensive knowledge of the various faculties of the mind, is desirable, as being cumulative, but this the ordinary physician, pressed by other and more frequently occurring duties, cannot acquire, so he must be content to occupy the position of an ordinary witness, declining to pass upon graver questions, on the plea of want of necessary study. In *Baxter* v. *Abbott*, 7 Gray, 71, it was held, "that a physician who had lived in the neighborhood of the testator, and had at times been his medical adviser, but who never had had charge of insane persons, except to advise that they be removed to an asylum, was competent to give an opinion as to the sanity of such testator." Again, it has been held, "that a physician who had not made insanity a special study, was not competent to testify as an *expert* in insanity upon a hypothetical case submitted to him, nor to give his opinion whether the prisoner, who had lived near him, and whom he had long known, but who had never been his patient, was competent to apply the rules of right and wrong." (*Com.* v. *Rich*, 14 Gray, 335.) In *State* v. *Windsor*, 5 Harr. 512, it was held, "that medical men in general were competent to express an opinion, on hearing all the evidence, as to the state of a prisoner's mind." On the other hand, (*Fairfield* v. *Bascomb*, *supra*), it seems that a physician "who had for more than thirty years given especial attention to the treatment of the insane, could not be admitted to testify, as an *expert*, as to the mental capacity of a person not previously insane, but in the last stages of disease."

"A singular decision was lately rendered in the Supreme Court of Massachusetts. The case under consideration was the pollution of Mystic Pond, the water supply of Charleston, by the influx of tannery refuse. Three chemical experts gave their testimony, among them Prof. Benj. Silliman. But Prof. Silliman, besides being a chemist, holds the degree of M.D., and his testimony in the latter capacity, as to the effect of decaying organic matter in water, on the health of those who drink it, was offered in court. It was, however, refused, on the ground that Dr. S. was not a practicing physician. The absurdity of the ruling becomes all the more apparent when it is known that Dr. S. has, for many years, been a professor of medical chemistry." (Galaxy.)

It is evident that the courts have not succeeded in establishing a positive rule as to who are experts.

A writer in the American Law Review, April, 1871, to whom I am indebted for many of the preceding citations, remarks farther:

"Titles, diplomas, occupations, and professional reputations, have no judicial weight or meaning, and are of use only so far as they serve as guides in particular cases. It is purely matter of discretion how much importance shall be attached to them."

He gives as a reason for these adverse rulings, that "Courts are continually forced to decide, on the spur of the moment, upon the pretensions and claims to credit, of persons offered as *experts*, often upon the most abstruse and difficult subjects."

In this day of "Eclectic" Universities, and degrees *in absentia*, I would suggest—believing that a *diploma* is some evidence of attainments—that the following questions be propounded the witness, under oath: Are you a graduate in medicine? and if so, did you obtain your degree by following out the course of study required by the majority of medical schools? and if so, were you examined upon those studies by the gentlemen whose names are upon your diploma?

The Hon. Emory Washburn, in a paper read before the American Academy of Arts and Sciences, (American Law Review, vol. 1, p. 61), says: "When a man is offered to testify as an expert, it is the duty and province of the judge to determine, upon evidence and inquiry, whether he answers to that description or not. Now, what is wanted in an expert is, first, that he should be sufficiently acquainted with the subject matter upon which he is called, to *know* what its laws are; and, in the second place, he should be honest and impartial enough to state his opinion or judgment upon these fully and fairly, so far as it can be done, and how they bear upon the issue to be tried. He should not be selected as the witness of this party or that, or to represent this view or that, of the matter in controversy. He should be like an arbitrator or a commissioner, who, standing indifferent between the parties, is to hear the facts which they have to offer; and then, by applying his own knowledge as a man of science, to form an opinion, and declare it, as a guide for the court and jury in forming a final judgment in the cause.

"I would, therefore, have experts selected by the court, as they appoint auditors to determine matters of account; or commissioners to take testimony, or to make partition of estates; or other like agents and instrumentalities in the administration of justice. Under such a rule, there would not, ordinarily, be any great difficulty in the parties themselves selecting who should be thus employed. And if there was, the very fact that one party or the other insisted upon a favorite witness, would be sufficient to put the court upon their guard in allowing him to act as such. The subjects upon which experts may be called are as numerous as the departments of business, and the transactions of life, in which laws and rules of action are applied. But the number of experts in any one department is ordinarily small, and easily distinguished. Who those are, in any district or region, and what are their relative character and fitness to act, can be ascertained by the court without any great difficulty, in each case as it arises. And though cases may occur where, by the selection or exclusion of one or another who may be named an expert, injustice may be done to a party, the great cause of justice will, on the whole, be vindicated, and the confidence of the public in the purity of its administration, as well as their respect for its ministers and instrumentalities, strengthened and confirmed."

Dr. Ray, (Contributions to Mental Pathology, pp. 422—424), after adducing many weighty reasons why experts should not be appointed by the Executive, says, in regard to their being selected by the court, he sees nothing in favor of *this* course, "beyond the chance of securing a higher degree of honesty and competence." Farther along he admits that the appointment of an expert by the Executive has its advantages, viz: "Skilled testimony on any subject, thus obtained, is free from the objections made against it as it is usually obtained. It is not given in the interest of one party or the other; it is unbiased by any pecuniary arrangements, and it escapes the damage of a cross-examination. It is the *well-matured* conclusions of a calm, deliberate, thorough investigation."

He favors the system in use in Germany and France, which is very similar to the one advocated by Professor Washburn. Dr. Ray qualifies his preference by saying:

"But it must be borne in mind that in Germany, for the most part, there is no trial by jury; and that in France a criminal case passes through its most decisive stages before it is submitted to a jury."

It has been said that neither "quacks" nor mere speculative theorists are to be received as experts. But who will draw the lines of distinction here? The Supreme Court of New York, in the case *Ex parte Paine*, 1 Hill, 665, tried to do it, and failed by not being comprehensive enough. It designated as such, "whoever offers to practice either homœopathy or allopathy, as his patients may wish;" which is very true, and yet such a person might make a very good *expert.* Why exclude him, when others are permitted, no more honest than he is? Inasmuch as the law recognizes no "schools," and it has been decided that a physician must be governed by the rules of that "school" he professes, (*Bowman* v. *Woods*, 1 Iowa, 441), it would seem that the testimony of experts, selected from one school, should not be received to impugn the professional rules and practice of practitioners of another "school." In reality there is but one "school," that whose rules require that the public "be enlightened, so that injuries may not be sustained by the unwary, from the devices and pretensions of artful empirics and imposters."

The right to object to any evidence as not proper to go to the jury, or as not being of legal credence, ought to be earnestly insisted upon. Thus, in an action for malpractice, the testimony of a "natural bone-setter" should be objected to, for the reason that a witness who swears falsely to matters of professional belief and opinion cannot be punished for perjury. Evidence in such cases does not and cannot extend beyond opinion and belief, and the cross-examination of such a witness would only tend still farther to enlist the prejudices of the jury in behalf of the "*poor plaintiff*" and "*abused*" witness. How much sanctity is there in an oath to one who is a fraud upon society, and who knows himself to be a fraud, as is the case with "bone-setters," "cancer doctors," etc.?

### CIRCUMSTANTIAL EVIDENCE.

Evidence as given by medical witnesses, acting as experts, is usually circumstantial or presumptive, that is, their evidence is deduced or inferred from one or more facts which are directly proved or admitted. It would seem indeed that nearly all evidence is of this character. A witness testifies to a certain act, he describes the manner in which the action was performed, he *presumes* it was done in earnest or in a jest, that is his *opinion*, and unless he is permitted to give this opinion as a part of his evidence, he fails to convey to the minds of the jury that which is sought for in his testimony. Upon a trial where intoxication is the subject in question, it has been held, "that the appearance which indicates intoxication cannot be so perfectly described in words as to enable persons not eye-witnesses to judge with accuracy on the subject."

In a case in which a mother was indicted for the murder of her child, in giving his charge to the jury, on the subject of evidence, Gibson, C. J., said:

"No witness has been produced who saw the act committed, and hence it is urged for the prisoner, that the evidence is only circumstantial, and consequently entitled to a very inferior degree of credit, if any credit at all. But that consequence does not necessarily follow; circumstantial evidence is, in the *abstract*, nearly, though perhaps not altogether, as strong as positive evidence; in the *concrete* it may be infinitely stronger. A fact positively sworn to by a single eye-witness, of blemished character, is not so satisfactorily proved as is a fact which is the necessary consequence of a chain of other facts sworn to by many witnesses of undoubted credibility. Indeed, I scarcely know whether there is such a thing as evidence *purely positive*. You see a man discharge a gun at another; you see the flash, you hear the report; you see the person fall a lifeless corpse, and you *infer*, from all these circumstances, that there was a ball discharged from the gun, which entered his body and caused his death, because such is the usual and natural cause of such an effect. But you did not see the ball leave the gun, pass through the air, and enter the body of the slain; and even testimony to the fact of killing is, therefore, only inferential, or, in other words, circumstantial. It is possible no ball was in the gun; and we *infer* that there was, only because we cannot account for the death on any other supposition. In case of death from the concussion of the brain, strong doubts have been raised by physicians, founded on appearances verified by the post mortem examination, whether an accommodating apoplexy had not stepped in at the nick of time, to prevent the prisoner from killing him, after the skull had been broken in pieces. I remember to have heard it doubted in this court, whether the death of a man, whose brain oozed through a hole in the skull, was caused by the wound, or a misapplication of the dressing. To some extent, however, the proof of the cause which produced the death rested on circumstantial evidence.

"The only difference between positive and circumstantial evidence is, that the former is more immediate, and has fewer links in the chain of connection between the premises and conclusion; but there may be perjury in both.

"A man may as well swear falsely to an absolute knowledge of fact as to a number of facts, from which, if true, the fact on which the question of guilt or innocence depends must inevitably follow. No human testimony is superior to doubt; the machinery of criminal justice, like every other production of man, is necessarily imperfect; but you are not, therefore, to stop its wheels. Because men have been scalded to death or torn to pieces by the bursting of boilers, or mangled by wheels on a railroad, you are not to lay aside the steam-engine.

"Innocent men have doubtless been convicted and executed on circumstantial evidence; but innocent men have sometimes been convicted and executed on what is called positive proof. What then? Such convictions are accidents, which must be encountered; and the innocent victims of them perished for the common good, as much as soldiers who have perished in battle. All evidence is more or less circumstantial, the difference being only in degree; and it is sufficient for the purpose, when it excludes disbelief—that is, actual and technical belief; for, he who is to pass on the question is not at liberty to disbelieve as a juror, while he believes as a man.

"It is enough that his conscience is clear. Certain cases of circumstantial proofs to be found in the books, in which innocent persons were convicted, have been pressed on your attention. Those, however, are few in number, and they occurred in a period of some hundreds of years, in a country whose criminal code made a great variety of offenses capital. The wonder is they have not been more. They are constantly resorted to, in capital trials, to frighten juries into a belief that there should be no conviction on merely circumstantial evidence. But the law exacts conviction, whenever there is *legal* evidence to show the prisoner's guilt, beyond a doubt; circumstantial evidence is legal evidence.

"If the evidence in this case convinces you that the prisoner killed her child, although there has been no eye-witness of the fact, you are bound to find her guilty. For her sake, I regret the tendency of these remarks; but it has been our duty to make them, and it will be yours to attend to them." (Elwell's Malpractice, 267.)

As an illustration of the value of this sort of evidence in detecting crime, and also to show some of the difficulties to be encountered by the medical expert, I subjoin the following history of the celebrated Parkman case:

Dr. Webster, the alleged murderer, had a residence in a Medical College in Boston. He requested Dr. Parkman to call on him at his rooms, on the 23rd of November, 1849, at which time and place they would discuss and settle a financial matter that existed between them. Dr.

Parkman was seen at about two o'clock to enter the Medical College, after that time he was never again seen alive. The prisoner stated that Dr. Parkman did not keep his appointment nor was he in the college that day. On the next Friday week, in the furnace, adjoining the prisoner's laboratory in the College building, there were found numerous bones mixed with the cinders and slag of the furnace, also blocks of mineral teeth. These blocks of teeth with the jaw, it seems, had been so placed in the furnace that the cold draft of air passing into the furnace, had prevented them from being as completely destroyed as were the other portions of the body. A quantity of gold which had been melted, was also found. In a vault there were found other bones, and in a tea chest there was found the entire trunk of a human body and other bones. These parts, when arranged in their proper positions, made a body of the size and apparent age of the missing man; besides, it presented the peculiarities belonging to Dr. Parkman. It being in a medical college it is likely that these parts would have been, in some parts, in duplicate, but such was not the case. However, with all this there would have been room to doubt, had it not been for the artificial teeth.

Here came in the expert evidence. Several dentists gave testimony to the teeth being those of Dr. Parkman. A mould had been made of the doctor's jaw, and this had been preserved. Its shape was such that the discovered block of teeth fitted perfectly. On the left side of the thorax a penetrating wound was discovered, and to this the death was attributed.

After a long and careful investigation the prisoner was found guilty. He subsequently made a confession, in which he stated that he first struck Dr. Parkman on the head with a heavy stick and then stabbed him with the knife found in the tea chest with the body.

In case of personal injuries medical evidence of the highest order should be procured. The following case llustrates the importance of the above rule. The case is sent the editor of the *London Lancet*, by Mr. PERFECT, a surgeon of Hammersmitt.

"It is now thirty years ago, that accidentally passing through Packhouse, Turnham Green, my attention was attracted to a mob of persons of the lowest order assembled around the door of that inn, who were very loud in their execrations against a certain person who was suspected of having murdered his brother ; in corroboration of which I was told that his bones were found near the premises where he formerly resided, upon view of which a jury was then sitting, after an adjournment from the day preceding. I found that two surgeons had been subpœnaed to inspect the remains, and I had no doubt but that every information as to their character had been obtained ; curiosity alone, therefore, induced me to make way into the room, where I found that the coroner, and I believe, a *double jury*, were sitting for the second day, and were engaged in an investigation which tended to show that a farmer and market gardener at Suttoncourt Farm had, a few years before, a brother living with him, who was engaged on the farm, but whose conduct was dissolute and irregular to a degree that often provoked the anger of his elder brother, and sometimes begat strife and violence between them ; that the temper of the elder brother was as little under control as the conduct of the younger, and, in fine, that they lived very uncomfortably together.

"One winter night, when the ground was covered with snow, the younger brother absconded from the house (for they both lived together), by letting himself down from his chamber window ; and when he was missed the ensuing morning, his footsteps were clearly tracked in the snow to a considerable distance, nor were there any other footsteps *but his own*. Time passed on, and after a lapse of some few years no tidings were heard of his retreat, nor perhaps have there ever been since. Some alterations in the grounds surrounding the house having been undertaken by a subsequent tenant, (for the elder brother had then left the farm), a skeleton was dug up, and the circumstances appeared so conclusive that one brother had murdered the other, that the popular clamor was raised to the utmost, and a jury impanneled to investigate the case.

"After listening attentively to these details, I ventured to request of the coroner to be allowed to examine the bones, which I found were contained in a hamper basket at the further end of the room, and I felt much flattered by his immediate compliance, for he desired the parish beadle, who was in attendance, to place them upon the table; and having himself disposed them in their natural order, I found that they represented a person of short stature, and from the obliteration of the sutures of the skull, and the worn down state of the teeth, must have belonged to an aged person. But what was my surprise when I reconstructed the bones of the skeleton, and found the lower bones of the trunk to be those of a female. I immediately communicated the fact to the jury, and requested that the two medical men who had before given their opinions might be sent for, one of whom attended, and without a moment's hesitation corroborated my report.

"I need not add that the proceedings were instantly at an end, and an innocent man received the *amende honorable*, in the shape of an apology, from all present, in which the coroner heartily joined. It has since been proved, beyond all doubt, that the spot where the bones were found was formerly the site of a large gravel pit, in which hordes of gypsies not only assembled, but occasionally buried their dead, and perhaps more skeletons are yet to be found in that vicinity."

It will not be in courts of justice alone that the medical man will be called upon to solve questions his peculiar studies alone enable him to do. These questions, the proper solution of which, if it do not advance him in the estimation of the community in which he lives, will at least prevent censure from being inflicted upon an honorable profession. A case in point is the following, which, as it illustrates the general feeling of anxiety that existed for several weeks after the disastrous Chicago fire of 1871, I will here give.

As soon as it was known to what gigantic proportions the fire had extended, the Rev. Mr. ——, hastened to Chicago to look after some friends then residing in the city. With some difficulty the site of his friends' residence was found. All that remained were the crumbling walls of the basement, partly beneath and partly above the level of the street. Amid the *debris* of brick and mortar were the springs of a bed, a sword and scabbard, and some charred and broken bones. These being together, what more natural thought than that they were the remains of — perhaps his friend or friends. The fragments were carefully collected and brought to the interior of the State. The then medical attendant of the gentleman was called and his attention directed to the basket of remains, with a request to determine what bones they were. Approaching with due reverence, for he supposed, himself, they were human, he set himself to the task. There were nothing *but* fragments ; however, he succeeded in arranging enough of them to give him something of an anatomical resemblance to the tuberosity of the ischium with its ramus, and the ramus of the pubes, but not those of a human being. His report was immediately given that the bones were probably the pelvic bones of some domestic animal. But how had they got there ? The reports then current concerning the frantic actions of animals in the streets during the fire, warranted the assumption that while the building was burning, some animal had rushed in at an open door or window and had there perished. Comparing the reconstructed fragments with the pelvic bones of a cow satisfied the Reverend gentleman that if his friends had perished he had failed to find their remains. The gentlemen were forced to accept the situation and await the developments of time. A letter containing an account of the discovery of the bones, and the fears they had awakened, was directed to such point as the family would be likely to have gone had they been spared. By return post a reply was received from the friend in question. It read: "*Dear Sir:* Myself and family are safe. The bones you speak of are probably the remains of a very fine pair of *antlers*, that I left in my library."

### EXAMINATION OF EXPERTS.

Much of the ill-report into which expert evidence has fallen depends upon, first, the false method of selecting expert witnesses, and second, upon the manner in which such witnesses are examined by counsel. This is often of a character not calculated to confer much respect upon the legal profession. This arises from two principal causes; first, counsel are too apt to suppose that the witnesses selected by the opposite party, are selected purposely, to render a false opinion in the case; and, secondly, they engage in the examination of such witnesses, without any knowledge of medical terms, whereby they might construct an intelligent inquiry. To this may be added, the witness is frequently called upon, without any opportunity for preparation, to answer questions which require thought for their proper solution. Time for this is not given. It is true he has his remedy in replying "I do not know." Physicians have no court of ultimate appeal; if they had, much of the censure so lavishly spent upon them, now, would be deserved. Then, too, if counsel would give as earnest thought to the development of truth, as they do to the gaining of their cases, the cause of justice would be more substantially subserved. "The best evidence the case will admit," is quite differently construed by lawyers and physicians, and for this, the mode of selecting *experts* is largely responsible.

In respect to the clashing of opinions among medical witnesses, gentlemen of the law are the last persons who should make uncharitable remarks, as the citations herein made abundantly show. That they give miserable testimony, in a miserable way, a recent ecclesiastical trial gives decided evidence. It should ever be kept in mind that opinions, formed from facts in evidence, are apt to be antagonistic. While the expert should be able to determine between the apparent and the real, his difficulties should not be enhanced by the unfair and ungenerous manner in which counsel frequently propound their questions. In fulfilling the functions of justice, courts should exert their power to compel counsel to put their questions clearly.

Opportunity is offered counsel to put his questions ambiguously, by requiring experts to answer upon hypothetical cases, or as to certain designated facts existing in the case, "supposing them to be true." Now the expert may not have been present when these facts were testified to, and if he was he would not be permitted to found his opinion upon them, for whether they be true or not, is for the jury to decide, and not the witness.

We find in practice so many points of dissimilarity in the same disease, modified, as each case is, by age, sex, temperament, concurring disease, social position of the patient, that there will necessarily arise, in the testimony of medical experts, seeming inconsistencies and contradictions that have no reality in fact. Under such circumstances the position and credibility of the witness stands in no enviable light.

Where a witness testifies to a fact which is wholly or partially the result of reason exercised upon particular circumstances, it is obvious that the reasons of the witness for drawing that conclusion are of the most essential importance for the purpose of substantiating that his conclusions are correct ones. These reasons the witness should be permitted to give.

The expert, finding his testimony called in question, may wish to fortify it by quotations from standard medical authorities. Whether he should be permitted to do this, authorities differ. In *Collier* v. *Simpson*, 5 Carr. & Payne, 73, Tindal, J., said: "I think I cannot receive medical books." It was further held, that although professional books or books on science are not admissible, witnesses might be asked the grounds of their judgment and opinion, which might in some degree be founded on those books as a part of their general knowledge.

Abbott, J., also, in *Rex* v. *Donellan*, objected to medical books in evidence: he said, "We cannot take the fact from any publication; we cannot take the fact as related by strangers."

"It appears to us that no witness can follow this advice without compromising the right and dignity of his profession, as well as the force of his evidence; for it would not be difficult to show that medical evidence altogether is little else than a reference to authority * * *. Is an oath more binding than the solemn act of sincerity between the author and the world by the very act of publication?" (Edinburg Med. and Surg. Journal, vol. 19, pp. 480, 610.)

In *State* v. *Rogers*, medical books were admitted, but in a subsequent case the same court refused to admit them. (Elwell's Malpractice, 331.) It was held, in the case of *Bowman* v. *Wood*, 1 Iowa, 441, that, as "medical witnesses were permitted to refer to and quote authors, there was no reason why they might not be permitted to read the views of distinguished authors. The opinion of an author as contained in his works is better evidence than the mere statement of those opinions by a witness, who testifies as to his recollection of these from former reading." So also in *Stoudenmeier* v. *Williamson*, 29 Ala., medical works, admitted or proved to be standard with the profession, are held admissible as evidence, with proper explanation of technicalities or phrases not generally understood.

The expert should not complain when the privilege of fortifying his testimony is denied him, as it is a rule applicable to lawyers as well.

Spencer, C. J., says of law books, that they "may be sometimes read to inform the mind of the court, but never as evidence."

"The medical witness, therefore, has no just grounds of complaint, because his books are not received in evidence. The court honors his individual opinion as of higher value than that of an outside author. The court presumes, that from reading these authors, close thought and actual observation and experience; the witness under oath, subject to a cross-examination, will more certainly enlighten the case than if it depends upon the published opinions of authors, who, perhaps, had a favorite theory to support or an old prejudice to influence them, on a question or subject constantly advancing. Then the author himself may have changed his opinions since the book was written." (Elwell's Malpractice, 335.)

The expert is to give an opinion founded upon the general principles of his science. It is right that his experience, his ability and his own sense of right should receive due acknowledgment at the hands of the court. But on the other hand all these circumstances are subject to great abuse. *Experience* and *gray hairs* are often appealed to; especially does the public adduce these as evidence of great ability. Of the two the gray hairs are often of the most value. In respect to experience, a writer has said, "I believe that no small portion of that odious discrepancy which has prevailed among medical witnesses, whereby the lustre of medicine itself has been tarnished, is chargeable to the prevalent affectation of being men of *experience* rather than men of *learning*."

It is usually such men whom we hear decrying medical authorities. It was in rebuking such a one, that the Hon. Chief Justice Dallas said, "I will not sit here and hear science reviled by ignorant tongues."

The medical witness may be asked if such and such authorities are good. To this general question, his acquaintance with medical writers should be such that he could give a general answer, but if he is asked if he agrees with such and such authority in matter of opinion, he should be quite certain what those opinions are before answering.

Bearing in mind that he is not to give an opinion as to the merits of the case, he is to answer all questions, that are permitted by the Judge, whether by counsel or jury, clearly and briefly, and if this is not sufficient to cover the entire truth, he is to add that which will.

Difficulties often arise between counsel and expert from the use of common words and terms which have a technical force. For instance, a phrase in very common use among physicians when speaking about what should be done in a given case, is, the "indications" are so and so. So also any symptom or occurrence, in the course of a disease, which serves to direct to a suitable course of treatment, is said to be "indications" for such treatment. Now this is a strictly medical use of the word, and is correct, yet counsel would generally object to it, and would ask what you meant by it, and when you replied that the symptoms, conjoined with your knowledge of the principles of medicine, directed to a proper treatment, counsel would be quite likely to curtly reply, "why don't you say so then." A recent writer in the American Law Review, in reviewing the Wharton trial, objects to the adjective "*fulminant*" in connection with cerebro-spinal meningitis, characterizing it as an "alarming adjective." For lawyers to do this is a "*tort*," for which an "*action*" ought to "*lie.*"

The use of terms of various significations according to the import most naturally given them by the two professions, "necessarily creates ambiguity, a condition usually aimed at by the counsel, whose effort frequently is to make the worst appear the better reason." (Ordronaux.)

In the use of medical terms lawyers are quite as apt to commit a solecism, as physicians are in the use of legal terms. Thus the reviewer in the Wharton trial speaks of "*urinic*" poison, when he intended *uræmic;* so also counsel have asked concerning "*dysuria*" in the fæces. Dr. Taylor remarks: "A judicious witness will avoid anything like a triumph over his examiner under such circumstances, and simply put him right."

If lawyers would seek the advice of an intelligent physician, before sending their briefs and abstracts to the printer, many of these mistakes would not appear in print.

"The expert," Dr. Percival remarks, "should use his best endeavors, that his mind be clear, and collected, unawed by fear and uninfluenced by favor or enmity.

"Manifestations of warmth and zeal beyond that which the occasion calls for, over-forwardness in testifying that which will benefit the party, and an ill-concealed reluctance in declaring that which tends to his prejudice, flippancy and levity of manner, coldness and apathy in describing injuries which would naturally excite a contrary feeling, indications of subtlety, artifice and cunning, are with a multitude of other tests for estimating the true character of a witness and the value of his testimony." (Starkie.)

Physicians when acting as experts should avoid, as far as possible, the use of Greek and Latin words, making use of these only when the vernacular does not express it as well. So, too, they should avoid the use of words from modern foreign languages, and words of a strictly technical meaning, so far as they can consistent with clearness. However, in cases where doubt might arise, in the use of the vernacular, the foreign word is to be retained, with proper explanation. The expert is to aim at a happy medium in this respect, making his testimony, either oral or written, comprehensible to the common mind, by explanation as the case may require. "Be the plainest man in the world," said Sir William Blizzard, "in a court of justice; never harbor a thought that if you do not appear positive you must appear little and mean ever after; many old practitioners have erred in this respect. Give your evidence in as concise, plain and yet clear a manner as possible; be intelligent, candid, open, and just, never aiming at appearing unnecessarily scientific; state all the sources by which you have gained your information. If you can, make your evidence a self-evident truth; thus, though the court may at the time have too good or too mean an opinion of your judgment, yet they must deem you an honest man. Never be dogmatic, or set yourself up for judge and jury; take no side whatever; be impartial, and you will be honest. In courts of judicature, you will frequently hear the counselors complain when a surgeon gives his opinion with any the least kind of doubt, that he does not speak clearly; but if he is loud and positive, if he is technical and dogmatic, then he is allowed to be clear and right. I am sorry to have it to observe that this is too frequently the case." (Beck's Med. Jurisp., vol. 2, 12th ed., p. 959.)

The expert is to remember that when he talks of "excited sensibility," "reflex action," "endemic disease," etc., both judge and jury are in the dark, these being terms with which both are entirely unfamiliar. It will reflect no credit, either, for the expert to speak of "the *nerves* being *relaxed*," the term being more applicable to the muscular than to the nervous system. He is to give his testimony as briefly and as much to the point as possible. He should give his evidence in such a manner as will convey the impression that he believes it himself. If there should be any doubt, it should be made known during the "direct examination." If he permits himself to become a partizan for either plaintiff or defendant he will most certainly receive a severe rebuke from the judge or from the counsel of one or the other party.

This impartiality is most difficult to exercise by a witness who has been in professional attendance on either of the parties, and who has been discharged by such party, because he did not look unfavorably enough upon the case to suit the intentions of the patient who purposed bringing a suit for damages. Such cases frequently occur in connection with injuries received in quarrels.

"Parties who desire a medical examination for legal purposes, in this country, usually hunt up the doctor or demand that he visit them at the appointed time. In either case, when there is a desire to deceive the physician, there is a crutch, without which the patient cannot walk. Or if he comes to the physician's office he brings his wife with him because he is so weak, also pills and other medicines he has been taking. Did we come suddenly upon such a person, we would find him at some gainful employment or enjoying some pleasure." (Caspar, vol. 4, p. 81.)

It is usually in charitable practice that cases arise where it is claimed there is incapacity to make a living. These cases are surrounded with extreme difficulty. On the one hand, it is evidently the physician's duty to guard the interest of the community that employs him; at the same time, there are undoubtedly cases of serious illness, which it is impossible for a physical examination to reveal. When the applicant is an able bodied person and has become an object of public care through his own dissipation, it would only be just to assume that he is a malingerer and treat him accordingly.

The medical practitioner is frequently applied to by parties seeking to evade public work such as road labor. Certificates of this character, it is unnecessary to say, should be free from technicalities so far as possible, compatible with fullness and completeness. It should contain complete *date* of its execution, place *where* examination is made, clear statement of the facts upon which the physician's professional opinion is based,—and he should take care that this basis is carefully made out,—a statement of the reasons why such certificate is required, the object for which it is to be employed, the statement of the patient or his relations as to his condition, and the personal observation of the physician as to the actual morbid phenomena observed.

It might be advisable to refuse all certificates except under requisition from ruling Boards, and never to give one on the request of the party concerned or his relatives. The advantage of this method is, that when the medical examiner waits for an official requisition, he has the power to see the patient when he least expects such a visit, and should the certificate be adverse to the applicant, much unpleasantness will be avoided. The examiner should under no circumstances grant a certificate *instanter* at the request of the applicant.

Farther, here, as in cases before the court, the expert should form his opinions from his own observation, and not from that of his colleagues acting at the same time as experts. Another rule is, that the strictest courtesy is to be observed towards other experts associated in the case. "Respect yourself, and the public will also." It is the duty of the expert to pay great deference to the judge; as for opposing counsel, their self-imposed importance may be safely ignored. Their object ever is to have the witness testify in their favor, and they will use every means to accomplish this. "If, however, the dictation of the judge is such that it is antagonistic to the latest and most approved theories upon the case, the expert should respectfully so state it." Thus where a judge, altogether gratuitously, said to the medical witness, who was testifying to the value of the *docimasia pulmonum* ("Hydrostatic test, a method of proof to which the lungs of a new-born child are subjected, for the purpose of detecting whether it has or has not respired after birth; in other words, whether it was born alive or dead." Dunglison's Med. Dict.), "You do not appear old enough to have seen the late Dr. Wm. Hunter, but you must know that he was one of the most eminent physicians of England, and that he has stated that no dependence whatever is to be placed on this test." The expert replied, "I am aware that was his opinion, but I entertain a different one, and I believe mine is now the received theory among medical men." (Dr. Gilman, in Beck, p. 959, vol. 2.)

The courts are extremely jealous whenever a medical witness assumes any of the functions of the Bench, yet how frequently do judges arrogate to themselves, as just shown, the functions of the "expert." Especially will this be noticed in connection with cases of insanity.

Doubts occasionally arise, in cases of alleged murder, whether the death has been occasioned by the wound or by the unskillful and improper treatment of the wound. In the *Stokes* case it was proven that "the bullet, on entering the abdominal cavity, caused the *first* perforation of the peritoneum; then passing through the omentum, causing four more perforations of the peritoneum; thence through two knuckles (convolutions) of the ileum, four more perforations of the peritoneum and four of the small intestines; thence through the mesentery, two more peritoneal perforations; thence through the sigmoid flexure of the colon—whether it was the ascending or descending portion, I know not—with two more perforations of the peritoneum, a final perforation of the latter on its pelvic reflection over the psoas magnus muscle. There were, therefore, four (4) holes or perforations of the small intestines, two (2) of the large, and fourteen (14) perforations or distinct wounds of the peritoneum. The missile then passed below the muscular fascicule of the psoas magnus, and following the course of that muscle over the arch of the pubes, without causing any injury whatever to the innominata or haunch-bone, lodged near the trochantea minor of the femur or thigh bone." (Peugnet, Gunshot Wounds of the Abdomen.) And yet it was thought that the medical treatment of Fisk had a great deal to do with his death, simply because it could not be proven that no man who had ever recovered from a gunshot wound of the abdomen did not receive as many wounds of the intestines and peritoneum as he did. To prevent certain dangers, that were certain to arise in a penetrating wound of the abdomen, Fisk's surgeons gave him an opiate, as doubtless those who now call in question the treatment would have done, but Fisk died *post hoc, ergo propter hoc.*

This was one case in which, I believe, the medical witnesses were not called upon to deliver learned dissertations upon "wounds," it being admitted by the defense that Fisk had really received these.

In respect to definitions, medical witnesses had better not attempt them, without they give them in the words of the *statutes.* Especially this should be the case with such words as "insanity," "wounds," "poisons," "deadly," etc. The following definitions of a wound, under ordinary circumstances, would be considered *good* definitions: "A recent solution of continuity in the soft parts, suddenly occasioned by external causes." "A solution of continuity from violence of any naturally continuous parts." "An external breach of continuity directly occasioned by violence." "An injury to an organic texture by mechanical or other violence." (Taylor's Princip. and Pract. Med. Jurisp.) Dr. Guy accepts the element "*solution of continuity*," and then divides wounds into two classes: first, such as are *without solution of continuity*, and second, such as are *with a solution of continuity.* To the first class he relegates *contusions*, *concussions*, *simple fractures*, *dislocations*, and *sprains.* To the second he consigns *incisions*, *punctures* and *lacerations*, *compound fractures*, and *gun-shot wounds.*" (Forensic Med.) "In Legal-Medicine, the term *wound* comprehends all lesions of the body." (Beck Med. Jurisp., vol. 2, art. "Wounds.")

"Where a prisoner had bit off the end of a finger, it was held, on a case reserved, that it was no wounding. So, when the nose has been bitten off, it has been held no wounding. Any kind of instrument is sufficient, but there must be some instrument used, to make it a legal wounding. A stone bottle, a hedge-stake, a gun, and even a shoe, if off or on the foot. A kick from a *bare* foot would not be a wounding. It has not been settled whether the teeth of a dog, which had been set to bite a person, can be considered as instruments within these statutes." (Elwell's Malpract. and Med. Evidence, 315.) "A solution of continuity in the soft parts, produced by some mechanical agent." (Dunglison's Med. Dict., Philadelphia, 1866.) "To constitute a wound, the external surface of the body must be divided." (Am. Jurist, vol. 18.) "In France the legal and medical definitions accord. By a wound, is understood every local effect on the body produced by acts of violence, or by the application of any caustic or corrosive agent." (Beck, vol. 2, 279, 280.)

These numerous citations are made to show what difficulties medical witnesses will have to meet, and, knowing them, may to some extent be prepared to meet them. If the witness could not give a definition of a wound in the words of the statutes, he should incline to that adopted in France, then qualify it by "slight," "severe," "dangerous," etc., the meaning of which would be left to the professional knowledge of the witness. It would not be sufficient for him to make a naked declaration, that the wound was "dangerous." "He must, if called upon, state to the court satisfactory reasons for his opinion." In legal acceptation, those wounds in which the danger is not *imminent*, would not be dangerous. Wounds of the thoracic or abdominal organs, of the large blood-vessels, or a compound fracture of the head, with depression of the fragments of the skull, could well be considered dangerous, because life would be in imminent peril. Wounds that are only liable to be dangerous, remotely or only in certain cases, would not be considered dangerous. Such wounds might be considered "serious." Other wounds might be considered as "slight," or "very slight," etc. Very correct opinions may be formed from a large reading of "good surgical authorities in relation to injuries of different parts of the body."

### CONFESSIONS.

Confessions of guilt may be sometimes made to a medical attendant. These must be free and voluntary on the part of the person making the confession. Under no circumstances is this to be obtained by threat, promise or inducement. The physician's duty is to make no comments, but reduce the person's words to writing, as soon as convenient or possible, read it to the person and obtain his signature, countersign it himself, and if possible have it attested by other witnesses.

It should be borne in mind that persons under the influence of disease have made voluntary confessions of guilt, which, when health has been restored, they make haste to deny. The mental condition, therefore, of persons making such confessions should be carefully ascertained.

### DYING DECLARATIONS.

"Death-bed declarations also are receivable in evidence, when made after all hope of recovery has been given up. It is presumed that such persons are induced to speak the truth, by as powerful a consideration as an oath. It is not necessary that he should *express* his convictions. It may be inferred from the nature of the injury, or from other circumstances of the case." (Guy's Forensic Med.)

Persons arraigned by such declarations may vindicate themselves in the same manner as they might if the statements were made under oath.

The medical practitioner's duty in the case is to reduce the statements to writing, put no leading questions, nor any questions, except such as are calculated to explain ambiguous statements, read it to the dying person, and if possible obtain his signature. If there be no time for this, the physician is to make memoranda of the statements, in the *identical* words of the person, immediately. These notes he is permitted to use in court to refresh his memory, when he comes to testify. He cannot use a

*copy* of such notes. The physician must also satisfy himself as to the mental condition of such person, noticing particularly whether he be "calm and collected or otherwise, and whether he is under the influence of any strong bias or undue feeling of resentment."

The medical man is to note any signs the dying person may make, for these, if intelligible, are also received in evidence. (Elwell, 318.)

If time permits, the physician may relieve himself of much of this responsibility, by sending for a magistrate to take the dying person's deposition. This course should always be adopted when feasible.

Medical men should discourage the bringing of suspected persons before one who is dying, in order that he may be identified. Surgeons of considerable experience are aware that in persons dying from hemorrhage, or from wounds implicating the brain, delirium is quite apt to occur, and that this delirium is not easily recognized by non-professional witnesses. The ability to prove an *alibi* has upon more than one occasion saved the reputation and lives of persons wrongfully accused.

The duty of a medical witness, when called to view a dead body, will be considered when we take up the subject of Inquests.

(To be continued.)

